# Molecular Characterization, Gene Evolution and Expression Analysis of the F-Box Gene Family in Tomato (*Solanum lycopersicum*)

**DOI:** 10.3390/genes12030417

**Published:** 2021-03-14

**Authors:** Fulei Mo, Nian Zhang, Youwen Qiu, Lingjun Meng, Mozhen Cheng, Jiayin Liu, Lanning Yao, Rui Lv, Yuxin Liu, Yao Zhang, Xiuling Chen, Aoxue Wang

**Affiliations:** 1 College of Life Science, Northeast Agricultural University, Harbin 150030, China; neaumfl@163.com (F.M.); yw12_630@126.com (Y.Q.); neaucmz@163.com (M.C.); zy13263696020@163.com (Y.Z.); 2 College of Horticulture and Landscape Architecture, Northeast Agricultural University, Harbin 150030, China; neauzn@163.com (N.Z.); neaumlj@163.com (L.M.); lvrui34324080@163.com (R.L.); yuxinliu1223@163.com (Y.L.); 3 College of Arts and Sciences, Northeast Agricultural University, Harbin 150030, China; 13040216@163.com (J.L.); yaoln888@163.com (L.Y.)

**Keywords:** abiotic stresses, cis-elements, expression analysis, F-box, gene evolution, tomato

## Abstract

F-box genes play an important role in the growth and development of plants, but there are few studies on its role in a plant’s response to abiotic stresses. In order to further study the functions of F-box genes in tomato (*Solanum lycopersicum*, *Sl*), a total of 139 F-box genes were identified in the whole genome of tomato using bioinformatics methods, and the basic information, transcript structure, conserved motif, cis-elements, chromosomal location, gene evolution, phylogenetic relationship, expression patterns and the expression under cold stress, drought stress, jasmonic acid (JA) treatment and salicylic acid (SA) treatment were analyzed. The results showed that *SlFBX* genes were distributed on 12 chromosomes of tomato and were prone to TD (tandem duplication) at the ends of chromosomes. WGD (whole genome duplication), TD, PD (proximal duplication) and TRD (transposed duplication) modes seem play an important role in the expansion and evolution of tomato *SlFBX* genes. The most recent divergence occurred 1.3042 million years ago, between *SlFBX89* and *SlFBX103*. The cis-elements in *SlFBX* genes’ promoter regions were mainly responded to phytohormone and abiotic stress. Expression analysis based on transcriptome data and qRT-PCR (Real-time quantitative PCR) analysis of *SlFBX* genes showed that most *SlFBX* genes were differentially expressed under abiotic stress. *SlFBX24* was significantly up-regulated at 12 h under cold stress. This study reported the *SlFBX* gene family of tomato for the first time, providing a theoretical basis for the detailed study of *SlFBX* genes in the future, especially the function of *SlFBX* genes under abiotic stress.

## 1. Background

The F-box gene family is one of the most abundant and versatile families in plants. [1,2]. In the process of biological development and evolution, organisms have possessed many kinds of regulatory mechanisms to respond to external environmental stimuli. Among them, the physiological processes regulated by the F-box gene family are particularly important. In the entire F-box protein family, the sequence of containing F-box plays an irreplaceable role in the plant’s growth and development [3,4,5,6,7]. The F-box protein family is very large, meanwhile their functions are also diverse. They are mainly distributed in eukaryotes which main take part in the protein degradation in eukaryotes. The process of F-box degradation of most foreign proteins is mainly through the ubiquitin-proteasome pathway (UPP). The ubiquitin-proteasome system is responsible for removing most abnormal peptides and short-lived cell regulators, thereby regulating many stress response processes [8]. The ubiquitin-proteasome system is an important post-translational regulation mechanism. the main proteolytic mechanism in eukaryotes is through this pathway [9]. Cells use this process to quickly respond the changes in intracellular signals and environmental stimuli. There are many unfavorable external environmental conditions affect the plant growth and development, such as drought, salinity, heavy metal stress, and cold [10]. In the study of *Arabidopsis thaliana*, a model plant and some other plants, it was found that the F-box gene family belongs to one of the most polymorphic supergene families, and their important role permeates almost every plant life cycle [11]. Therefore, the F-box protein plays a key role in the growth and development of plants and the regulatory response to the living environment and endogenous signals.

The F-box was first discovered at the N-terminus of cyclin F [12]. F-box mostly functions as the SCF (SKP1-Cullin-F-box) complex. F-box contains a domain (containing about 40–50 amino acids) at the N-terminus, which is used to bind other components of the SCF complex [13]. The C-terminus is a highly variable domain (protein-protein interaction site), including LRR (Leucine rich repeats), Kelch, and WD40 [13,14]. The SCF complex consists of four subunits. The three subunits Cullinl/Cdc53, Rbxl/Rocl/Hrtl, and Skp1 together form a skeleton, which can specifically bind to different F-box proteins. Then the complex protein could recognize different substrates. Therefore, the F-box protein determines the specificity of substrate recognition in the recognition process [15,16,17]. According to the analysis of its gene structure, the number of exons and introns in the F-box genes are high variability, which just shows that these genes in plants are extraordinary. Generally speaking, the same type (not exactly the same substrate) can accept the regulation of some F-box genes with highly similar structure. Some F-box proteins may have highly similar functions. Most F-box genes in plants are involved in the regulation of many biological processes, allowing cells to quickly respond to changes in intracellular and extracellular signals and the continuous changes in the living environment. So the structure and function of F-box proteins research is necessary [8].

In earlier studies, *Arabidopsis* is an important plant carrier for scientists to obtain a lot of information about F-box genes. In plants, only 23 F-box proteins (18 of which are from *Arabidopsis*) are known for their functions and widely used in research. Some abiotic stresses such as cold, drought and salinity are important restrictive factors that affect plant growth and development [13,14]. Therefore, in order to keep normal physiological activities, plants must adapt to or resist these adversities at different physiological and molecular levels. The F-box protein encoding gene family plays a very important role in plant stress resistance. In recent years, many studies on F-box protein’s involvement in plant stress response have surfaced [18]. There are more than 43 different types of F-box proteins in monocot rice that can cope with different types of environmental stress, and through repeated database searches, 687 potential F-box proteins have been identified [19]. Most of them may play a positive regulatory role in adversity. Others, including *Piper nigrum*, *Phaseolus vulgaris* and *Triticum aestivum,* have similar expression results [20,21,22].

When the plants are exposed to abiotic stress, a series of morphological, physiological and biochemical changes will occur, such as slow growth, chlorophyll reduction and even flower and fruit drop, which will lead to crop failure. Cold and drought are two of the main abiotic stress factors, which seriously affect plant growth and production. In order to resist cold or drought damage, it is possible to cultivate abiotic-resistant and plant types. Cold and drought resistance are affected by multiple genes location. Therefore, it is necessary to dig out more genes related to abiotic resistance. In order to have better understanding of the mechanism of abiotic stress response, we focused on the F-box protein. F-box protein is mainly involved in the abiotic stress response of plants. The F-box genes of many miRNAs involved in the regulation of *Arabidopsis* growth and development may respond to cold and drought. Under low temperature stress, the expression of *miR393*, *miR397b* and *miR402* were increased, but the translation process of its target gene (putative F-box protein) is reduced. So the growth rate of plants is slowed down, which can improve the cold resistance of plants [23]. *MAIF1* is an F-box gene in rice that regulates *MiRNAs* and abiotic stress. Studies have shown that *MAIF1* participates in a variety of signal pathways to regulate the growth and development of rice roots. The gene *MAIF1* mainly through increasing the number of root cell divisions to establish rice abiotic stresses tolerance (such as chilling injury) [24]. *SlEBF3*, a new F-box gene in tomato, participates in affecting fruit ripening by interacting with the EIL (Ethylene-insensitive) protein in tomato and mediating its degradation [25] Another F-box gene, *ACIF1*, has been reported to have a positive regulatory effect on the Ve1-mediated response of verticillium dahliae and white rot of tomato [26]. These results are dedicated to provide useful information for further research on the role of F-box genes in plant abiotic resistance. This study identified the F-box gene family in tomato, and analyzed the molecular characterization, gene evolution, conserved motif and promoter regions. In addition, this study have analyzed the expression patterns based on the transcriptome data, and ten *SlFBX* genes were selected to validated the transcriptome’s result by qRT-PCR. The study will provide a theoretical reference for the research of F-box genes on plant abiotic resistance.

## 2. Materials and Methods

### 2.1. Plant Materials and Treatment

The tomato variety Glamour was planted in the growth chamber of the Northeast Agricultural University, with a light intensity of 120 μM photons m^−2^ s^−1^, photoperiod 16 h, day/night temperature 22/18 °C, stress treatment with cold and drought, the methods for stress treatment are shown in Zhou et al. [27], 100 mM salicylic acid (SA) and jasmonic acid (JA) were used to treat the 27-day-old plants, each solution was sprayed evenly over each blade and stop spraying before the solution drips from the leaf. The first fully expanded leaf from the top of the plants was collected with three replicates after 0 h, 6 h, 12 h, 24 h, 36 h and 48 h per treatment [27]. Three plants of the same growth were selected for each treatment as three biological replicates, and then three technical replicates were performed for each biological replicate.

### 2.2. Identification the Members of SlFBX Gene Family in Tomato

The Hidden Markov Model (HMM) of F-box domain (PF00646) was downloaded from the Pfam database (http://pfam.xfam.org, 1 December 2020). The DNA sequence, CDS (Coding DNA Sequence) file, total proteins sequence and the gff3 file were downloaded from the Ensembl Plants database (1 December 2020, http://plants.ensembl.org) and the genome annotation version is SL3.0. Using the hmmsearch tool from HMMER v3.0 to search domains similar to F-box domain in the total protein sequences of tomato [28], and set the value to 1 × 10^−5^. Then filtering the result with the cutoff value was set to 0.001. Besides, the full-length protein sequences were submitted to the Pfam, SMART (http://smart.embl-heidelberg.de, 1 December 2020, and CDD (Conserved Domains Database, 1 December 2020, https://www.ncbi.nlm.nih.gov/Structure/cdd/cdd.shtml) databases to confirm the candidate genes contain the F-box domain.

### 2.3. Sequence Analysis of SlFBX Genes in Tomato

The length, isoelectric point, and molecular weight of SlFBX protein sequences were analyzed by Bioperl and the ExPasy website (1 December 2020, https://www.expasy.org). The MEME-v4.12.0 software was used to identify the motif of SlFBX protein sequences [29], the main options of MEME software is followed: sequence use protein alphabet; maximum number of motifs to find was 10; the minimum and maximum motif width were 6 and 20. The gene structures were extracted from the gff3 file, then use the TBtools to statistics and visualize the motifs and exon-intron and UTR (Untranslated Region) regions. In order to identify the type of cis-elements in the promoter from *SlFBX* genes, 1500 bp of the genomics sequence upstream of the transcriptional start site was submitted to the PlantCARE website (1 December 2020, http://bioinformatics.psb.ugent.be/webtools/plantcare/html).

### 2.4. Phylogenetic Analysis and Chromosomal Location

The full-length sequence of certain members in *SlFBX* gene family from tomato was alignment by MUSCLE in MEGA version 7 software [30]. Build a neighbor joining tree with the bootstrapping was performed with 1000, the other parameters are default values. The location of all *SlFBX* genes and the length of chromosome were obtained from Ensembl Plants database and mapped by the TBtools [31]. In addition, in order to study the evolutionary relationship between *SlFBX* gene and other species, *F-box* genes in *Nicotiana attenuata* (NIATTr2), *Solanum tuberosum* (SolTub_3.0) and *Coffea canephora* (AUK_PRJEB4211_v1) were identified by the method in Section 2.2, and the evolutionary tree was drawn, and use iTOL (1 December 2020, https://itol.embl.de) for beautification. The genomes and protein sequences of these three species were download from Ensemble plants database.

### 2.5. Duplication, Ka/Ks and Synteny Analysis

Synonymous substitution rate (Ks) and nonsynonymous substitution rate (Ka) can reflects the evolutionary relationship between genes [32]. The blastall tool was used to blast the CDS sequences of 139 *SlFBX* genes, and the expectation value is 1e-20, the other parameters are default values [33]. Then faidx the result from blastall by samtools, and filtered the result used for calculate Ka/Ks value by a perl script. The criteria for filtering were as follows: the length of aligned genes was greater than 70% of the longer gene, and the similarity between the two genes was greater than 70%; the distance between the two genes was less than 100 kb [34]. Calculating the value of Ka and Ks with KaKs_calculator [35]. The formula T = Ks/r was used to calculate the divergence time, with r being the rate of divergence for nuclear genes from plants. For dicotyledonous plants the r was taken to be 1.5 $\times 10^{-8}$ synonymous substitutions per site per year [36].

Genes duplication and synteny were analyzed by MCScanX-transposed [37], the main option of is followed: MATCH_SCORE: 50, MATCH_SCORE: −1, MATCH_SIZE: 5, E_VALUE: 1 × 10^−5^. The inhouse script duplicate_gene_classifier was used to classify all pairs of *SlFBX* genes in tomato, including segmental, tandem, proximal, and dispersed duplications under the default criteria. Circos software was used to visualize collinear regions and members of gene families in the genome [38].

### 2.6. Expression Analysis Based on RNA-Seq

In order to research the expression patterns of *SlFBX* genes in tomato, the RNA-seq data were downloaded from NCBI (National Center for Biotechnology Information) database with under the accession number of SRP156535 at https://www.ncbi.nlm.nih.gov/sra/SRP156535 (1 December 2020). Control (SRR7652565, SRR7652566, SRR7652567), use cold instead of low temperature (SRR7652564, SRR7652570, SRR7652571), drought (SRR7652563, SRR7652568, SRR7652569). HISAT software was used to map the reads from all samples to the tomato genome [39]. The featurecounts tool of Rsubread package in R was used to the genes quantification [40] and visualized by TBtools.

### 2.7. RNA Extraction and Real-Time PCR Validation

Total RNA was extracted from the tomato leaves with plant RNA mini kit (Watson, China) and reverse transcribed to cDNA with the TransScript One-Step gDNA Removal and cDNA Synthesis SuperMix Kit (Applied Biosystems, Shanghai, China). Two genes were selected from each subtribe, a total of ten genes from *SlFBX* genes were selected to validate the accuracy of the RNA-Seq data. The primers as shown in Supplementary Table S1, were designed on NCBI and used for qRT-PCR. Each reaction contains 10 µL Unique AptamerTM qPCR SYBR Green Master Mix (Applied Biosystems, Shanghai, China), 0.4 µL forward primer (10 μM), 0.4 µL reverse primer (10 μM), and 1.0 µL of diluted cDNA sample. Finally, add sterile ultrapure water to replenish to 20 µL. The polymerase chain reaction (PCR) conditions were as follows: initial denaturation at 95 °C for 5 min followed by 40 cycles of denaturation at 95 °C for 10 s, 60 °C for 20 s and 72 °C for 20 s. The relative expression level was calculated using the 2^−ΔΔct^ method [41]. The actin gene was employed as a standardized internal control, and the relative mRNA levels in untreated normal plants were normalized to 3 biological replicates were employed for each sample. qRT-PCR data were analyzed by SPSS software (1 December 2020, https://www.ibm.com/cn-zh/analytics/spss-statistics-software).

## 3. Results

### 3.1. Identification of F-Box Genes in the Tomato Genome

A total of 166 putative F-Box genes were identified in the tomato genome by using HMMER software. In order to identify whether those genes contain F-Box domain, the proteins’ sequence of the putative F-Box genes were submitted to SMART, Pfam and CDD three databases. After removing the redundant sequences without an F-box domain, a total of 139 genes were obtained for subsequent study. They are named *SlFBX1* to *SlFBX139* according to the chromosomal location of the genes. Supplementary Table S2 shows the genes’ name, genes’ id, genes’ location, coding amino acid lengths, and isoelectric points (pI) of the 139 tomato *SlFBX* genes. As shown in Supplementary Table S2, the amino acid length of its coding protein is between 35 (SlFBX50) and 1662 (SlFBX117). Isoelectric points of those proteins ranged from 4.16 (SlFBX105) to 10.38 (SlFBX12). SlFBX have a wide range of molecular weights from 4079.9 Da (SlFBX50) to 189118.1 Da (SlFBX117). The results showed that the *SlFBX* gene family had many members, and the differences among the members, such as the length of the coding amino acid, isoelectric point and molecular weight, were all significantly different.

### 3.2. Motifs Identification and Gene Structure Analysis of SlFBX Gene Family

**Figure 1:**
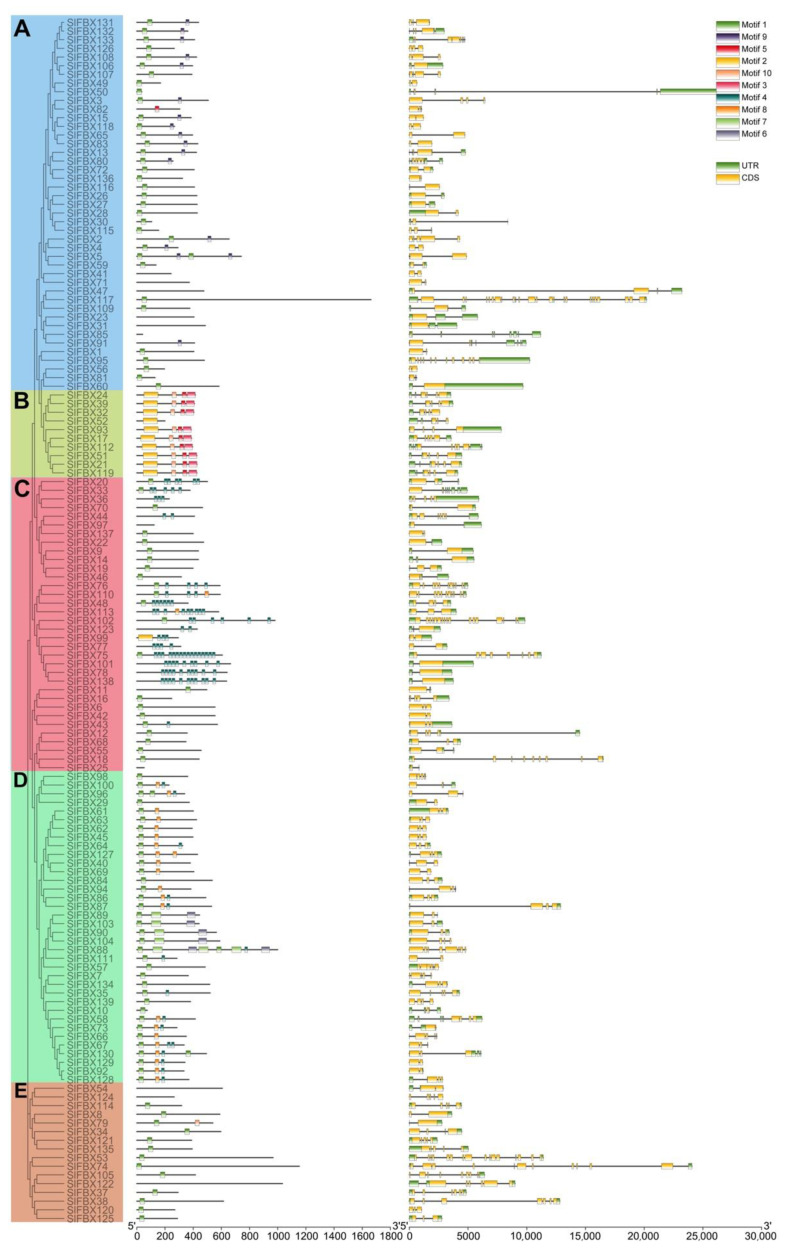
Phylogenetic relationships, conserved motifs of SlFBX proteins and structures of *SlFBX* genes in tomato. **A**–**E** represent different sub-families of *SlFBX* genes.

In order to determine whether the structure and conserved motifs of *SlFBX* genes are related to phylogeny, 139 *SlFBX* gene protein sequences were submitted to MEGAX software for analysis and used the MEME tool for conservative motif analysis. In addition, the extracted structural information of *SlFBX* gene family members was from tomato genome annotation files. Finally, we used the TBtools for visualization. Phylogenetic tree, motifs, and gene structures were shown as in Figure 1, respectively.

As shown in Figure 1, to clarify the phylogenetic relationship of *SlFBX* genes in tomato, 139 *SlFBX* genes were divided into 5 subtribes A–E after analysis by MEGA7.0 software, which contained 43, 10, 34, 36 and 16 *SlFBX* family members respectively and the evolutionary relationship of *SlFBX* genes was demonstrated.

There were 10 kinds of motifs with high confidence identified from *SlFBX* gene family, but about 7.9% (11/139) of the genes could not identify them, probably because the E-value of these 11 *SlFBX* genes were greater than that of other motifs. The sequences logos of all motifs are shown in Figure S1. Motif 1 appeared nearly 77% (107/139) of the time in all protein sequences, making it the most frequent motif of all motifs. There is a high correlation between the distribution of motif and phylogeny. For example, in subtribe A, motif 1 and 9 always appeared together, and motif 2, 3, 5 and 10 always seemed to appear together in subtribe B. Most of motif 4 are concentrated in subtribe C, and in subtribe C, SlFBX75 was the most motif 4 containing protein, and there were 16 motif 4 in SlFBX75.

However, the result show that there was no significant correlation between gene structure and phylogenetic relationship, and the distribution of introns from 1 (such as *SlFBX49*) to 24 (*SlFBX117*) was irregular.

### 3.3. Phylogenetic Analysis of SlFBX Gene Family

**Figure 2:**
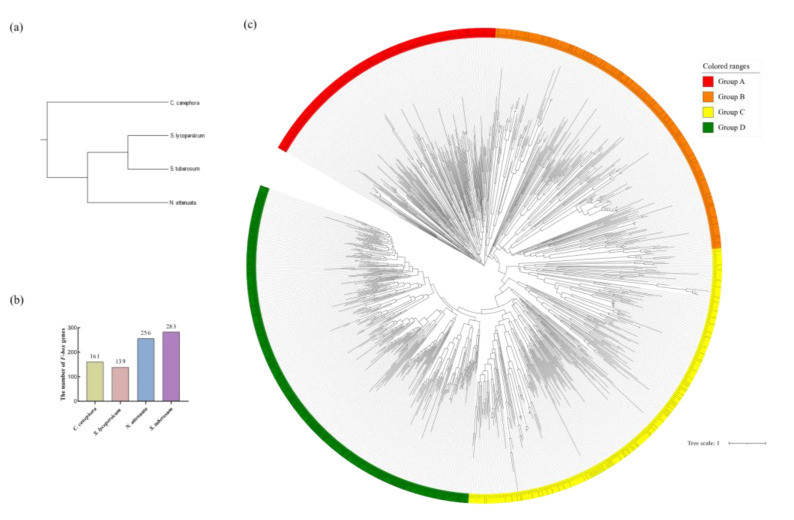
Phylogenetic relationship of F-box gene family. (**a**) Phylogenetic tree of 4 species. (**b**) Number distribution of F-box members in 4 species. (**c**) Phylogenetic relationships of F-box proteins identified in *S. lycopersicum*, *N. attenuata*, *S. tuberosum* and *C. canephora*.

In order to explore the phylogenetic relationship of *F-box* gene family, a total of 839 *F-box* genes were identified in *S. lycopersicum*, *N. attenuata*, *S. tuberosum* and *C. canephora*. And a species tree was built based on their CDS sequence, as shown in Figure 2a. There are 161 *F-box* genes in *C. canephora*, 256 *F-box* genes in *N. attenuata* and 283 *F-box* genes in *S. tuberosum* (as shown in Figure 2b). Based on the full-length of the protein sequence from 839 *F-box* genes, a phylogenetic tree was drawn as shown in Figure 2c. It can be seen that 139 *SlFBX* genes were randomly distributed in 4 groups (43 *SlFBX* genes in group A, 41 *SlFBX* genes in group B, 29 *SlFBX* genes in group C and 26 *SlFBX* genes in group D). According to Figure 2a, the 4 species have a difference in phylogenetics, but none of all *F-box* genes showed significant species differences, which to some extent reflected that the evolutionary relationship of tomato *F-box* genes was relatively conservative. *SlFBX* genes were produced before the differentiation of these species.

### 3.4. Promoter Cis-element Analysis

**Figure 3:**
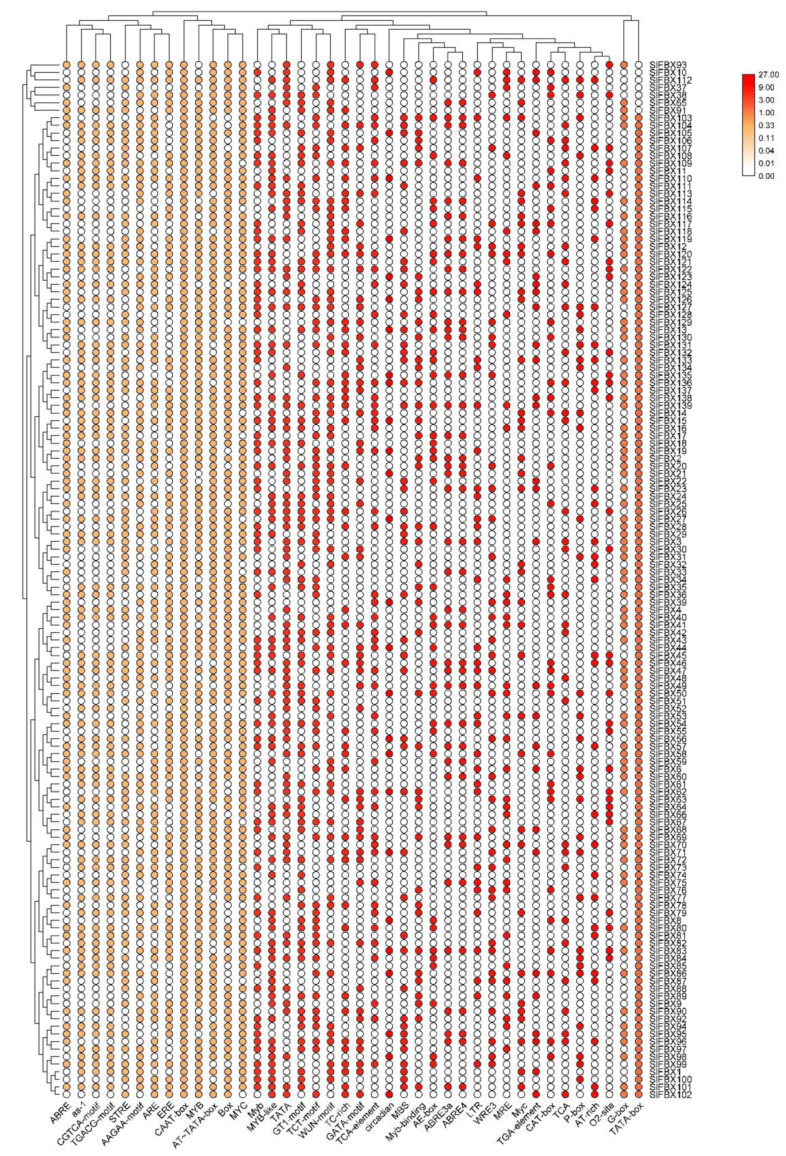
The cis-element of promoter in *SlFBX* gene family. The depth of the color represents the number of times cis-element occurs.

The 1500 bp sequence upstream of the *SlFBX* genes was selected and submitted to the PlantCARE database for the analysis of promoter cis-element. Statistics of all these promoter cis-elements and their frequency in all *SlFBX* genes were shown in Supplementary Table S3. As shown in Figure 3, some cis-acting elements with unknown functions or less frequent occurrences (less than 20) were removed, and then TBtools were used to create a heatmap visualization. These cis-elements are associated with plant hormones and environmental responses. Such as wound-responsive element (WUN-motif), methyl jasmonate (MeJA) responsive elements (TGACG-motif), the MeJA-responsiveness (CGTCA-motif), light responsive element (GT1-motif), anaerobic induction element (ARE), stress-response element (STRE), cis-acting element involved in the abscisic acid responsiveness (ABRE), ethylene-responsive element (ERE) and low temperature response (LTR element). In addition, there are nine cis-acting elements that appear only once in all *SlFBX* genes and, although these nine cis-acting elements appear very infrequently, they still have important functions such as the auxin-responsive element (AuxRE).

### 3.5. Chromosome Location Analysis

**Figure 4:**
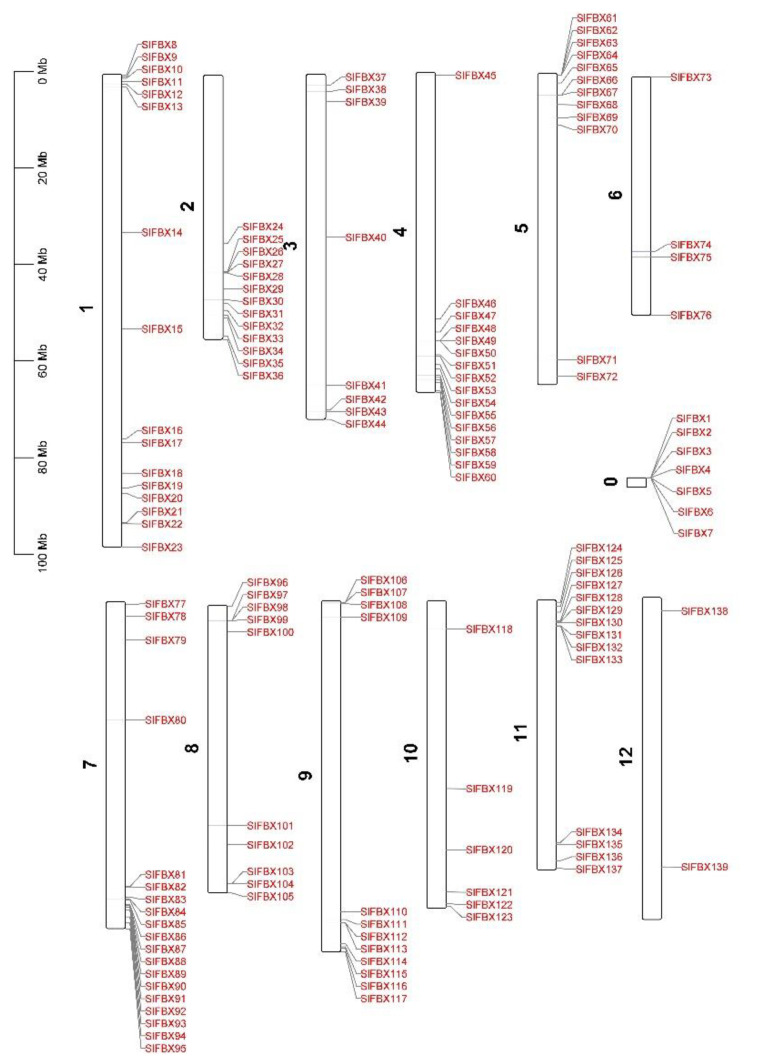
Chromosomal locations of *SlFBX* genes in tomato, 1 to 12 is the tomato’s 12 chromosomes, with 0 representing the location of genes not located on the chromosome.

In order to explore whether the *SlFBX* gene family is distributed regularly, the chromosomal locations of all *SlFBX* genes were mapped. The location of genes on chromosomes is shown in Figure 4. Except that *SlFBX1* to *SlFBX7* cannot be located on any of the 12 chromosomes of tomato, the remaining 132 *SlFBX* genes can be located on chromosomes. Except that there are only 2 *SlFBX* genes on chromosome 12, there are more *SlFBX* genes distributed on other chromosomes. In particular, 19 *SlFBX* genes are distributed on chromosome 7. At the same time, most of the genes are located far away from the centromeres, which may also indicate that gene replication is more likely to occur in loosely arranged regions of chromatin.

### 3.6. Expansion and Evolutionary Analysis

**Figure 5:**
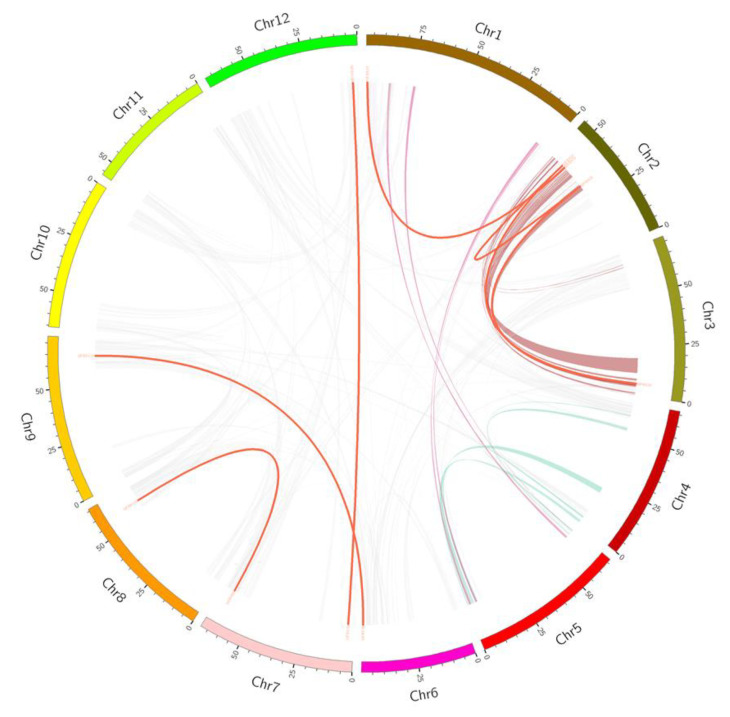
The syntenic pairs of tomato *F-box* genes from different duplication mode diagrams. The syntenic pairs from whole genome duplication (WGD) were linked by red lines. Purple, green and brown represent syntenic regions with large segments. The others syntenic pairs genes were shown with grey lines.

Gene duplication plays an important role in the evolution of the genome; it may accumulate evolutionary raw materials in the process of replication, thus contributing to plant evolution [42]. In particular, whole genome duplication (WGD) and tandem duplication (TD) have been found to be important for the expansion of many multigene families [43]. Therefore, this study also analyzed whether there were TD among 139 *SlFBX* genes, so as to explore the evolutionary relationship between *SlFBX* genes. There are 21 *SlFBX* genes have a replication relationship in *SlFBX* gene family as shown in Table 1. Six pairs of genes (ten genes) had TD. And 11 genes of *SlFBX* genes had WGD, six genes had transposed duplication (TRD) relationship, only four genes had proximal duplication (PD) relationship. The values of Ka/Ks range from 0.1586 to 1.8618. However, only four pairs of genes had Ka/Ks greater than one, and the rest were all less than one, indicating that most of the replication-related *SlFBX* genes were purifying selection. The earliest differentiation occurred in 73.9437 million years ago. The position of these WGD genes in the genome is shown in Figure 5.

### 3.7. Expression Analysis of SlFBX Genes Based on RNA-Seq

**Figure 6:**
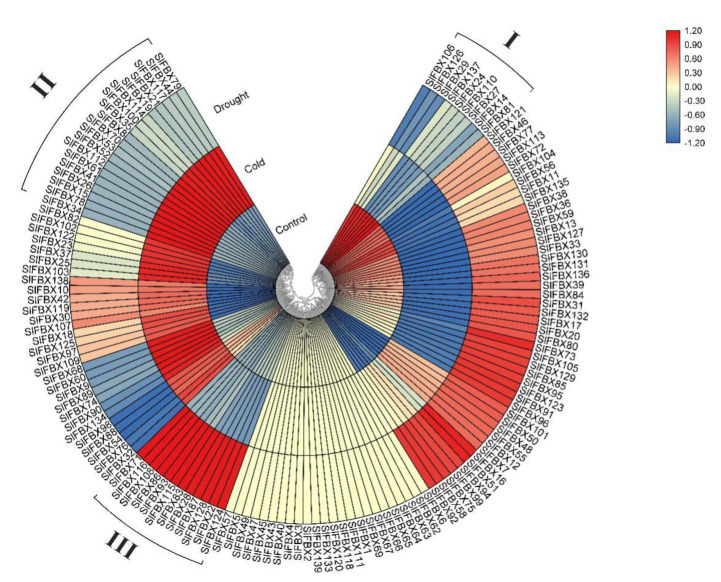
Transcriptional profiles of *SlFBX* gene family members in tomato under elevated cold stress and drought stress. blocks with colors indicate low/down expression (blue), high/up expression (red), and non-expression/no change (yellow).

Three groups of RNA-Seq data including tomato, treated with low temperature (cold), drought and without treatment (control), were used for this analysis. All of reads from the RNA-Seq data were mapped to the reference genome, 139 *SlFBX* genes were mapped under control, cold stress and drought stress. About 82.7% (115/139) *SlFBX* genes have a different expression in three groups of RNA-Seq data. All of the 139 genes were seen to have unique expression patterns under different conditions, but there are 24 *SlFBX* genes were not expressed in any of the three RNA-Seq data, as shown in Figure 6. Meanwhile, some genes showed an up-regulated expression trend in a single group and showed down-regulated or inactive expression in other groups. For example, the group I in control, the group II in cold and the group III in drought had a high up-regulated expression patterns only in their respective groups. The different expression patterns of all *SlFBX* genes also indirectly revealed the functional diversity of *SlFBX* gene family in tomato growth and development.

### 3.8. Expression Analysis of SlFBX Genes under Stress Conditions

**Figure 7:**
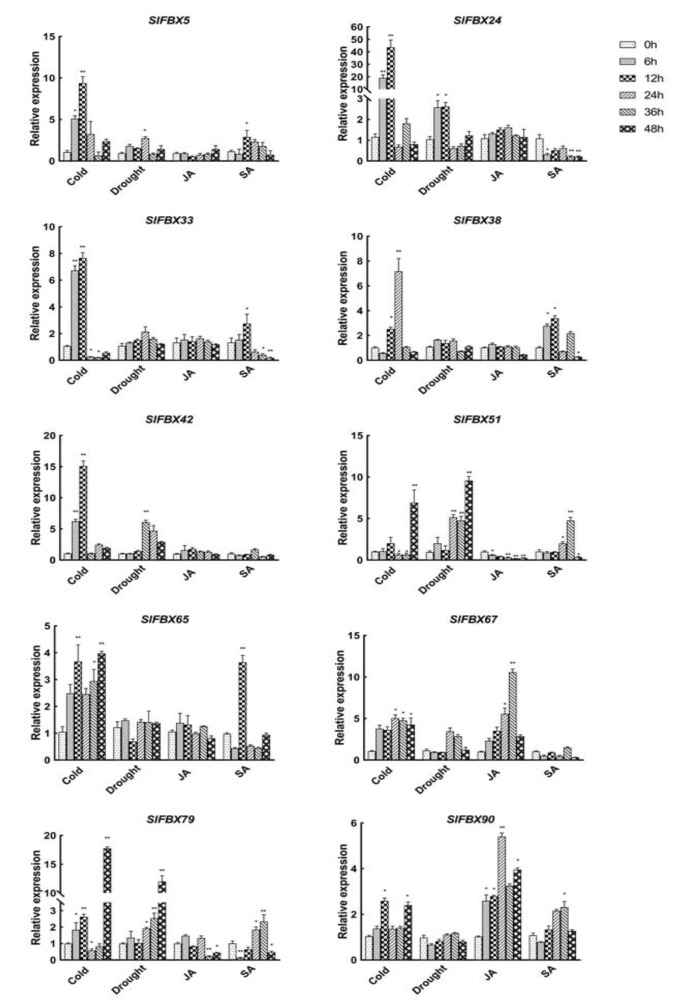
Relative expression analysis of 10 genes in the *SlFBX* gene family under cold stress, drought stress, JA treatment and SA treatment in tomato. ANOVA was used to test significance (*p* < 0.05 indicates significant difference, indicated by *; *p* < 0.001 indicates high significance, indicated by **).

In order to verify the accuracy of RNA-Seq data and to investigate the response of the *SlFBX* gene family in tomato to abiotic stress (cold and drought) and hormones treatment, this study selected two *SlFBX* genes from each phylogenetic subtribe, a total of 10 genes, namely *SlFBX5*, *SlFBX24*, *SlFBX33*, *SlFBX38*, *SlFBX42*, *SlFBX51*, *SlFBX65*, *SlFBX67*, *SlFBX79*, *SlFBX90*. qRT-PCR detected the expression of the 10 members of the *SlFBX* gene family. The results in Figure 7 reveal that the expression of *SlFBX51, SlFBX65* and *SlFBX79* was significantly up-regulated under cold stress, especially, *SlFBX79* expression was up-regulated about 20 times after 48 h of cold stress. *SlFBX5*, *SlFBX67* and *SlFBX90* also tended to be up-regulated after 48 h of cold stress. However, *SlFBX24* and *SlFBX33* showed an up-regulated trend only at 6 h or 12 h of cold treatment but showed an obvious down-regulated trend after 24 h.

The expression of *SlFBX79* and *SlFBX51* also increased significantly under drought, and they have similar expression patterns under drought. The expressions of *SlFBX51* and *SlFBX79* were up-regulated 10 times and 12 times respectively under drought stress at 48 h. In the first 24 h of drought stress, *SlFBX5*, *SlFBX24* and *SlFBX42* showed a trend of up-regulated expression, but their expression decreased gradually with the passage of time. The remained genes showed no obvious up-regulated or down-regulated under drought.

For the expression of 10 genes under JA treatment, *SlFBX51*, *SlFBX65* and *SlFBX79* showed a relatively obvious down-regulated trend. *SlFBX67* and *SlFBX90* showed a trend of up-regulated expression within 48 h, but *SlFBX67* showed a significant up-regulated expression at 36 h, and the expression at 48 h was less than that at 36 h, but still higher than 0 h. The expression level of *SlFBX90* after JA treatment were more than twice that of the control group (0 h). For SA treatment, all of the genes except *SlFBX90* showed a downregulated expression trend after 48 h, *SlFBX90* was also only a slightly up-regulated expression.

## 4. Discussion

Abiotic stress seriously affects the growth and development of plants. In the long process of evolution, plants have produced many physiological and biochemical mechanisms to resist these stresses. Tomato is one of the most important vegetable crops in the world [44]. However, its resistance to abiotic stress is relatively weak [45]. When tomatoes are grown in cold or drought conditions, the growth and development of tomato will be seriously affected, and even lead to death [46]. As one of the super families in plants, F-box family plays a very important role in many aspects of plant growth and development [47]. In previous studies, many F-box gene families have been identified in plants, such as *Oryza sativa* [19], *Malus* [48], *Pyrus bretschneideri* [49], *Hordeum vulgare* [50], and *Zea mays* [51]. Plant’s F-box protein has many members and complex functions. At present, the function of F-box has mainly in the aspects of hormone response and photomorphogenesis. In the ethylene signaling pathway, F-box protein negatively regulates the ethylene pathway [52]. F-box protein is also a positive regulator of gibberellic and auxin signaling pathways [52,53,54]. However, there have been few studies on the *F-box* gene family of the tomato.

In this study, 139 members of the *SlFBX* gene family in tomato were identified, the sequence characteristics and structures of *SlFBX* genes, conserved motif, phylogenetic relationships, cis-elements of promoter regions, tandem duplication and whole genome duplication were obtained. In addition, expression patterns of 10 *SlFBX* genes were explored based on the data of transcriptome and validated the result by qRT-PCR.

As shown in Supplementary Table S2, the average molecular weight of members of the SlFBX gene family is 48.6 KDa, the average pI is 7.66, close to neutral pH.

In plants, different F-box proteins bind to SCF subunits to recognize different substrates. The domain at the C-terminal of the F-box protein is the determinant of binding to different SCF subunits. F-box protein plays a role in a variety of signal transduction pathways, such as stress resistance, photomorphogenesis, and hormone response [55,56]. Kelch repeats, LRR domains, WD40 domains and FBA domains were identified in the *SlFBX* gene family in tomato. These domains have important functions in many aspects of plant growth and development. SlFBX14, SlFBX14, SlFBX22, SlFBX46, SlFBX60, SlFBX79, SlFBX98 and SlFBX137 contain the Kelch structure, and the number of Kelch varies from 4 (SlFBX137) to 16 (SlFBX79), Kelch repeats domain participates in the circadian clock regulation process and recognize substrates and mediates their ubiquitination. [57,58]. SlFBX36, SlFBX48, SlFBX75, SlFBX94, SlFBX99, SlFBX101 contain the LRR domain. The disease resistance of LRR has been extensively studied in plants, such as *S. lycopersicum* [59], *Oryza sativa* [60] and *Arabidopsis* [61]. LRR recognizes flagellin (flg22) in the defense responses in *Arabidopsis* [62], and priming the plant resistance [63]. Seven WD40 domain has identified in SlFBX18, WD40 domain not only promote protein interaction [64], but also participate in the flavonoid biosynthesis in *Arabidopsis* [65], *Perilla frutescens* [66], *Zea mays* [67], *Oryza sativa* [68], *Medicago truncatula* [69] and *Vitis vinifera* [70]. There are about 29.3% (27/139) of SlFBX proteins containing the FBA domain, the FBA domain is involved in carbohydrate metabolism [71] and in signal transductions [72] in *Zea mays* [73], *Arabidopsis* [65], *Spinacia oleracea* [74], *Nicotiana tabacum* [75], *Sesuvium portulacastrum* [76] and *S. lycopersicum* [77]. Although these domains are not all of the structural domains of *SlFBX* genes, it can be seen that *SlFBX* genes play an important role in many aspects of plant growth and development. 

In all 139 *SlFBX* genes, 137 genes were identified *SlFBX* cis upstream of the promoter function components were shown in Figure 3. There are 0 cis-element in *SlFBX5* and *SlFBX7*, inaccuracy in genome assembly and annotation may be the reason why cis-elements were not obtained in *SlFBX5* and *SlFBX7*. So, improving the optimization of existing genome for scientific research and production practice is of significance. There are three category functions of cis-element: growth and development (such as G-box) [78], hormonal response (such as ABRE) [79] and stress response (such as LTR) [80]. All cis-elements of *SlFBX* genes are shown in Supplementary Table S3.

New genes were produced with the gene duplication if a mutation occurred, and WGD/WGT events could explain why the F-Box family has so many members and functions in a variety of plants. Members of the *SlFBX* gene family have various functions. Then, 139 *SlFBX* genes were divided into five subtribes according to the full-length sequence of proteins as shown in Figure 1. *SlFBX* genes in the same subtribes are highly conserved in protein sequence, so we can find that the genes have the TD relationship are all divided into the same subtribes, such as the subtribe A, which contains tandem duplication genes *SlFBX131*, *SlFBX132* and *SlFBX133*. Meanwhile, according to Table 1, there are PD and TRD relationships in the *SlFBX* gene family, which gives new impetus to the increase of the number of *SlFBX* genes and their differentiation.

The phylogenetic relationships of 839 *F-box* proteins were shown in Figure 2, *S. lycopersicum*, *N. attenuata* and *S. tuberosum* are members of Solanaceae, and *C. canephora* is close to the Solanaceae family. Although not all four plants belong to the Solanaceae, they do not show a relatively long evolutionary distance in terms of *F-box* genes’ evolution. For example, in group A, the *S. lycopersicum*’s gene *SlFBX123*, the *N. attenuata*’s gene *OIT01487*, the *C. canephora*’s gene *CDP15407* and the *S. tuberosum*’s gene *PGSC0003DMT40006975* are all on the same branch, and they are very close in evolutionary distance. Meanwhile, WGD (or whole genome triplications, WGT) plays an important role in the evolution of *F-box* genes. Combining Figure 2 and Figure 4, group A, group B and group C have the WGD genes in Figure 2c, such as *SlFBX*89 and *SlFBX* 103 in group A, *SlFBX*138 and *SlFBX*78 in group B, *SlFBX*76 and *SlFBX*110 in group C, there are none of the WGD or WGT genes in group D. But there are only two pairs of PD genes that were all in group D, which may indicate that PD genes of the other three species are mainly concentrated in group D, based on structural similarity. In addition, the number of *F-box* gene in *S. lycopersicum* was much smaller than that in *S. tuberosum* and *N. attenuata,* which may indicate that only a small amount of *F-box* gene was copied in WGD or WGT events in *S. lycopersicum*, which leads to this result. In a word, the results not only indicate that the evolutionary relationship of *F-box* genes in the four species is very close, but also prove that the *F-box* family, as one of the largest superfamilies in plants, is very widely distributed in the four plants. WGD or WGT play an important role in the expansion of *F-box* gene family. Meanwhile, based on the phylogenetic relationship shown in the results, we speculate that the function of *F-box* genes in other species is similar to *SlFBX*. In other words, these *F-box* genes maybe have similar functions in abiotic stress, hormonal response, or photomorphogenesis.

This study revealed that *SlFBX* genes could respond to four different treatments—cold stress, drought stress, JA treatment and SA treatment. This indicates that *SlFBX* can response to four kinds of treatments, but *SlFBX* expression levels are also different. In Figure 6, the expression of *SlFBX* genes was similar between the control group and the drought group, but different from the cold group. For example, *SlFBX121* showed an up-regulated expression trend in both the control group and the drought group but showed a down-regulated expression in the cold stress group. Ten *SlFBX* genes were selected for qRT-PCR, and the results of qRT-PCR showed that the expression of most of the genes was consistent with the results obtained from transcriptomic data analysis. However, there is a subtle difference. For example, the expression of *SlFBX5* can be detected in qRT-PCR, but it is not shown in transcriptomic data. Although *SlFBX5* does not show significant difference in expression under different stress, the possible reason is that there are some errors in transcriptomic data measurement. This study found that *SlFBX51* had a significantly up-regulated expression under both cold stress and drought stress, and a significantly up-regulated expression under SA treatment at 36 h, but showed a significantly down-regulated expression at 48 h. Moreover, the expression change of *SlFBX24* was the most significantly induced by cold stress. At 12 h, *SlFBX24* was up-regulated nearly 50 times, but the expression after 24 h was not significant compared with that at 0 h, which may indicate that *SlFBX24* responded quickly in the early stage of cold stress and played an important role. In addition, this study showed a different tendency compared with the RNA-seq data from Zhou’s (2019) study [25]. In transcriptional analysis, *SlFBX79* showed upregulation only in the cold stress group, but the qRT-PCR results showed that *SlFBX79* also showed an up-regulated trend under drought, although the upregulation multiple was smaller than that under cold.

## 5. Conclusions

In this study, a total of 139 *SlFBX* genes were identified by using bioinformatic methods in tomato. Gene structure, chromosomal location, phylogenetic relationship, duplication events, and expression based on transcriptome data were analyzed in detail. In addition, ten genes were selected and qRT-PCR was used in order to verify the accuracy of transcriptome data and to explore the true expression of *SlFBX* under abiotic stress. The 139 *SlFBX* genes were divided into five subtribes according to their protein sequences, which have various structures and functions. In the phylogenetic analysis of the four species, all the F-box genes showed close evolutionary relationships. There were 30 genes with replication relationships, indicating that gene replication plays an important role in the generation and functional differentiation of gene families. This is the first study to confirm that *SlFBX79* plays an important role in drought and cold, providing a basis for further study on the role of *SlFBX* gene family in tomato breeding and resistance improvement.

## Figures and Tables

**Table 1:** The duplication pairs genes of *SlFBX* gene family in tomato.

Duplicate 1	Duplicate 2	E-Value	Mode	Ka/Ks	Time(Mya *)
*SlFBX52*	*SlFBX32*	1 × 10^−99^	TRD	0.7449	8.8202
*SlFBX48*	*SlFBX78*	9 × 10^−11^	TRD	1.3014	34.7772
*SlFBX101*	*SlFBX78*	0	TRD	0.1443	73.9437
*SlFBX102*	*SlFBX78*	4 × 10^−13^	TRD	1.1327	37.7539
*SlFBX23*	*SlFBX31*	2 × 10^−36^	WGD	0.8398	34.3571
*SlFBX24*	*SlFBX32*	0	WGD	0.2099	18.9381
*SlFBX24*	*SlFBX39*	0	WGD	0.1586	20.7803
*SlFBX32*	*SlFBX39*	0	WGD	0.1820	19.4613
*SlFBX76*	*SlFBX110*	0	WGD	0.1879	25.0713
*SlFBX138*	*SlFBX78*	0	WGD	0.2358	23.4021
*SlFBX89*	*SlFBX103*	0	WGD	0.5185	1.3042
*SlFBX88*	*SlFBX89*	4 × 10^−106^	TD	1.8618	5.9052
*SlFBX131*	*SlFBX132*	0	TD	0.9049	6.3030
*SlFBX132*	*SlFBX133*	1 × 10^−120^	TD	0.9152	12.4690
*SlFBX62*	*SlFBX63*	0	TD	0.4898	13.1833
*SlFBX63*	*SlFBX64*	2 × 10^−89^	TD	0.6093	19.6296
*SlFBX66*	*SlFBX67*	2 × 10^−111^	TD	0.5459	19.4655
*SlFBX106*	*SlFBX107*	8 × 10^−154^	PD	1.0846	7.2143
*SlFBX26*	*SlFBX27*	4 × 10^−118^	PD	0.5952	20.0988

* Mya means million years ago.

## Data Availability

Data is contained within the article or supplementary materials.

## Supplementary Materials

The following are available online at https://www.mdpi.com/2073-4425/12/3/417/s1, Figure S1: The motif of SlFBX protein sequences; Table S1: Primers for qRT-PCR; Table S2: Base information of *SlFBX* gene family; Table S3: Cis-elements information.
