# Supplementary figures and images for: Molecular Characterization, Gene Evolution and Expression Analysis of the F-Box Gene Family in Tomato (Solanum lycopersicum)
Source: Genes (Basel). 2021 Mar 14;12(3):417. doi: 10.3390/genes12030417 (PMC7998346; doi:10.3390/genes12030417)

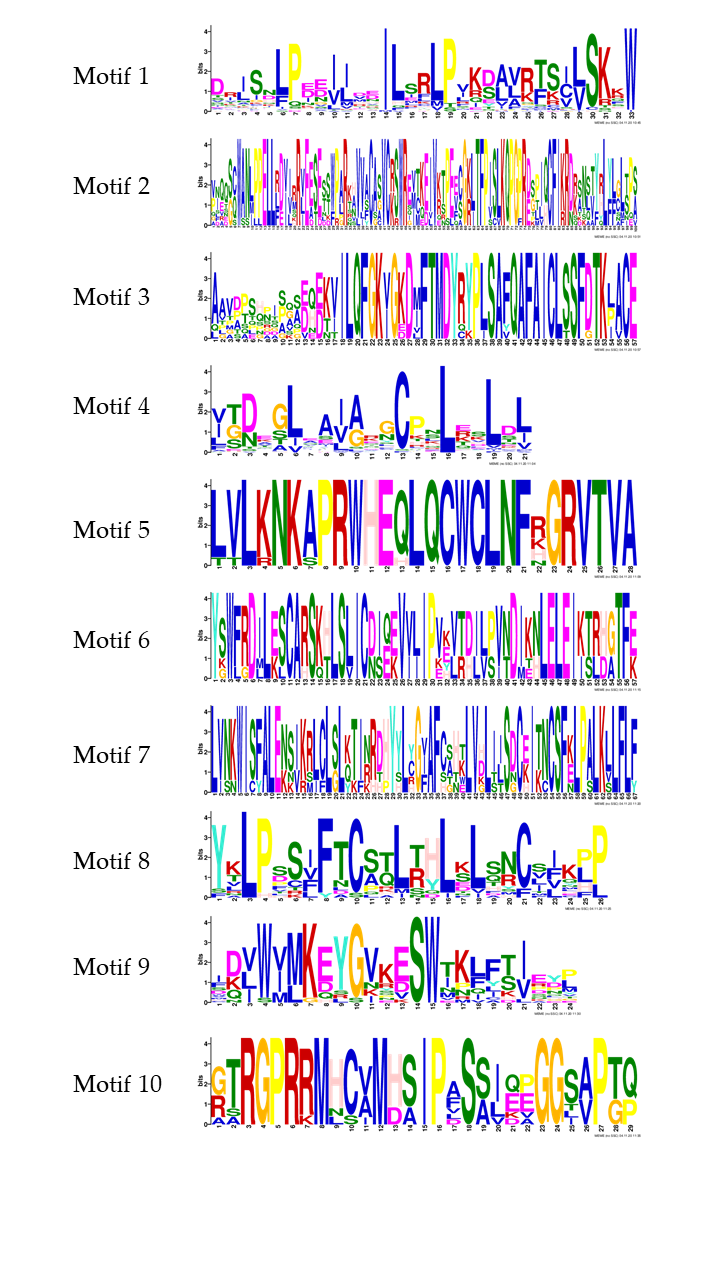

Supplement: Supplementary file 1 [file genes-12-00417-s001.zip › S.Fig 1.tif]
